# Co-representation breaks down beyond the dyad in UK adults

**DOI:** 10.1371/journal.pone.0318545

**Published:** 2025-02-25

**Authors:** Sophie J. Milward, Jamie Whitehouse

**Affiliations:** 1 University of Portsmouth, Portsmouth, United Kingdom; 2 Nottingham Trent University, Nottingham, United Kingdom; Universite Clermont Auvergne, FRANCE

## Abstract

Cooperation is so deeply embedded in human psychology that we spontaneously track a partner’s task as well as our own when acting in a pair. This automatic ‘co-representation’ of a partner’s mental representation of their task has been argued to be key to the sophisticated social coordination we see in human adults. However, our day-to-day encounters are not limited to one-to-one interactions. This is the first published study to investigate co-representation in groups, with results from a group Joint Simon task suggesting that co-representation may break down in groups larger than two. Exploratory analyses also suggested a complex interplay between spatial and social relationships between individual members within a group. We propose a novel hypothesis based on these findings: when we lack the capacity to track everyone in a group, we may be able to selectively track those who are the most salient or relevant. This provides key information about the limits of our capacity to keep others in mind, and the psychological underpinnings of how we do so.

## Introduction

On a day-to-day basis, we work with others to achieve goals that are not achievable on our own. One key mechanism that is argued to aid us with this is ‘task co-representation’, whereby actors automatically track their partner’s mental representation of their task role as well as their own. Existing work has only studied this phenomenon in dyadic interactions, such as carrying an item with the help of a friend or having a one-to-one conversation with a colleague [1,2]. However, we regularly need to coordinate with an entire group of people, such as moving a heavy sofa with three friends, or navigating a work meeting with a group of colleagues. The current study breaks this mould by investigating the influence of up to five co-actors in a minimally joint task and exploring how spatial and social inter-relationships influence task tracking.

Individuals have long been known to struggle to ignore irrelevant information (e.g., stimulus location) when asked to respond to target information (e.g., colour) in the Simon task [3]. Typically, participants see, for example, a red or green ring on a finger, which points to one of two response buttons: green or red. They respond more quickly to green rings when the finger points towards the compatible green response button than towards the incompatible red button. This effect vanishes when the participant is only required to respond to one variation of the target feature (e.g., green stimuli) but not the other (e.g., red stimuli)[2], suggesting it is the representation of both response options (green and red) and their corresponding spatial relationship (left and right) that causes interference in the original, two-choice version. Joint action research has found that when this task is shared between two co-actors (e.g., I respond to green, you respond to red), compatibility effects return, similar to when an individual has to represent both responses her/himself [2]. The authors of this finding argue this is best explained by co-actors ‘co-representing’ their partner’s task in the same way as if they were performing it themselves.

Task co-representation has gained popularity and has been investigated under a wide range of contexts and with several populations [4–24]. However, it has never been extended beyond the dyad. This is a limitation not only because group settings make up a considerable proportion of our day-to-day interactions, but also because there are theoretical implications for testing the limits of co-representation. This mechanism has been argued to be automatic [2–25], meaning that it occurs quickly, and without much cognitive effort. If this is the case, multiple co-actors should be automatically represented relatively effortlessly. However, this has recently been called into question in the field of ‘automatic’ visual perspective-taking. New evidence suggests that perspective-taking is limited to one other perspective [26], which implies that it requires cognitive effort to represent additional perspectives (although see [27]) and therefore does not fulfil the criteria for automaticity [28]. It is therefore important to put task co-representation to the test in the same way, by identifying whether there are limits on the number of co-actors that can be represented.

Further, there are theoretical reasons to expect specific limits on the number of co-actors that can be represented at once. In the field of recursive mindreading, studies have shown that the majority of individuals can perform higher-order levels of intentionality up to four levels (I know that Janet knows that John knows that Kate knows), but few can achieve five levels [29,30]. This is consistent with naturalistic work on conversation in groups, in which we see the ‘Dinner Party Problem’ [31], whereby groups tend to break off into smaller groups once they exceed four members. This study will test whether task co-representation also shares this limit of four (three co-actors’ tasks, plus one’s own).

Additionally, some have argued that Joint Simon effects are not the result of mental representation of a partner [2], but rather of using the partner as a non-social point of reference to code response options as ‘left’ and ‘right’ (Referential Coding Account [32]). Some have argued that referential coding can only occur when there is a salient co-actor present within one’s peripersonal space: the area directly around one’s body within arm’s reach [9]. These researchers argue that physical proximity provides a (sometimes misleading) cue indicating collaboration, which results in coding of the partner’s actions even when there is no explicit joint goal. Research manipulating the distance between co-actors has found mixed evidence for the influence of peripersonal space on Joint Simon effects, with two studies suggesting the effect does not occur for co-actors in extrapersonal space [9–33] and one suggesting it does [10]. However, none of these studies directly compared set-ups where actors were in peripersonal versus extrapersonal space within the same experiment. By testing the Joint Simon task in group settings, we can directly test the influence of physical proximity not only within experiment, but within-subjects, by varying the distance between group members within the same task.

Finally, existing work on co-representation has found mediating effects of the relationship between the two co-actors in a joint task [6,34–38]. If co-representation is limited to a certain number of co-actors, there are two options for how this plays out in larger groups. One possibility is that co-representation simply breaks down completely, so that the individual does not track anyone. Alternatively, the individual may selectively track certain individuals within the group (up to their capacity) and ignore others. If the latter is true, there must be some mechanism in place for selecting which members of a group should be tracked and which should be ignored.

This study utilizes the Joint Simon task to measure interference effects from acting alongside more than just one partner. We adapted the task to allow up to five co-actors to participate at once and compared performance on compatible and incompatible trials (indicating interference effects) in groups with between 2–5 co-actors and solo participants. We predicted a limit, whereby co-representation would occur in groups of 4, but not 5, based on evidence from recursive mindreading and natural conversation. We also manipulated the physical distance between co-actors, to help distinguish between different theoretical accounts of joint task interference. An interference effect for peripersonal partners but not extrapersonal partners would support a referential coding account, whereas effects regardless of physical distance would support a co-representation account. Finally, we measured inter-individual relationships in order to explore the potential mediating role of social dynamics between individuals within group settings.

## Method

### Participants

254 adults were recruited from the undergraduate student population at the University of Portsmouth, UK and from local events in the Portsmouth area (including events for lower income communities) between 26^th^ August 2022 and 15^th^ February 2023. Ethical approval was granted by the University of Portsmouth Science and Health Faculty ethics committee (SHFEC 2022-009A) and all participants gave written informed consent. Undergraduate students were tested in a lab on campus and participants at events were tested in either a gazebo outside or a quiet part of a community centre. Participants were paid £10 or 1 course credit. Individuals were allocated to each of the 5 between-subjects conditions (Group Sizes 1–5), depending on the number of sign-ups at different times across the testing session (see S1 for distribution across locations and Group Size condition). After excluding 54 participants (recruited from a music festival) for reporting alcohol use prior to participation, a total of 200 participants remained in the dataset (*age*: mean =  28.22, standard deviation =  16.25, range from 18–80 years, *sex*: 143 female, 50 male, 10 undisclosed; *self-described gender:* 142 female, 50 male, 11 undisclosed, *self-described ethnicity*: see S2 ). An a priori power size calculation (GPower [39]: *f* = .25, *α* = .05, power = .8) indicated 196 participants suitable for this design.

### Design

A 2(Compatibility) x 5(Group Size) mixed design was used, where participants were allocated to groups of 2–5 co-actors, or alone, and completed both Compatible and Incompatible trials (randomly presented). Incompatible trials were further divided according to Distance between co-actors, where Peripersonal trials showed the finger pointing towards a co-actor sitting within the target actor’s peripersonal space, and Extrapersonal trials showed the finger pointing towards a co-actor outside of this space.

### Materials

**Fig 1:**
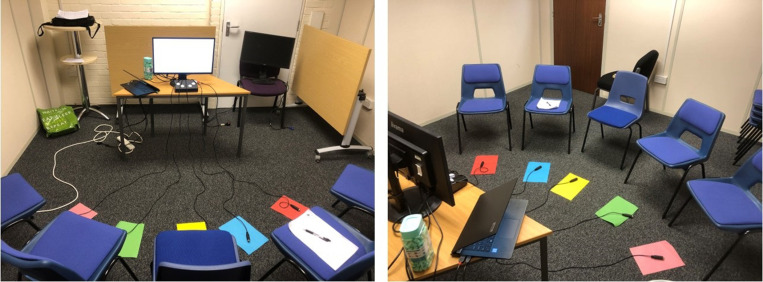
Task set-up in the lab. Set-up was identical at events, but either in a 3m x 3m gazebo or a quiet, screened off area of a community centre. Chairs were placed with the front edges of the seat 20 cm away from one another, so that actors sitting directly next to one another would be within arm’s distance (Peripersonal space) but those sitting further away would not (Extrapersonal space). Participants were seated 120 cm from the screen, in a semi-circle.

**Fig 2:**
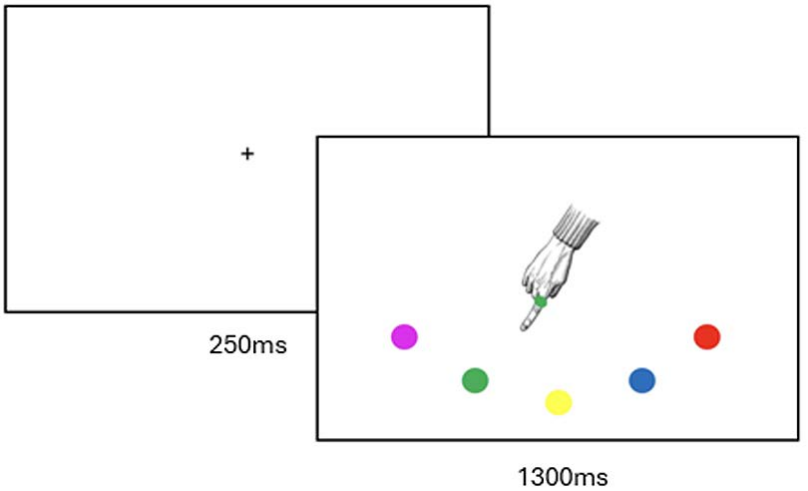
Stimulus presentation sequence for a single trial. Duration of screen presentation displayed below screen shots. The example shows a Compatible trial, where green is the correct response. For incompatible trials, the finger does not point towards the correct response, but rather to one of the other colours. Peripersonal trials are where the finger points towards the response option directly next to the correct response (yellow or pink in this example), compared to Extrapersonal trials where it points towards a more distant response option (e.g., blue or red).

A Joint Simon task was programmed in EPrime 2.0 [40]. The task set-up and stimulus presentation sequence are shown in Figs 1 and 2 respectively. The task consisted of a practice block of 10 trials (5 Compatible, 5 Incompatible), followed by 3 blocks of 150 trials, with a short (estimated < 30 second) break between each block. Participants were instructed to call the experimenter back into the room when the break appeared on the screen, at which point she would ask if participants were ready to keep going. Once all participants confirmed, she set the next block running. Within each block, rings of each of the 5 colours were presented an equal number of times, pointing towards each of the 5 response options an equal number of times. Consequently, 1 out of 5 trials was Compatible, and each response option was correct an equal number of times. Participants were asked to insert foam earplugs before starting the task, to avoid any perceptual information from the sound of the button presses. Buttons were discrete, hand-held USB clickers (*The Black Box Toolkit*, 2003) held in the participants’ laps, so movement made from pressing the button was minimal.

### Procedure

Participants were randomly allocated to one of the 5 seats and corresponding colour response. For groups smaller than 5, the set-up remained identical (i.e., 5 chairs and 5 ring colours), but the seat and colour responses allocated to participants were counterbalanced across each level of Group Size. Participants were instructed that they would soon see a hand with a ring on the finger and that they should press their button as fast as possible whenever the ring is the same colour as the piece of paper on the floor in front of their chair. Full instructions can be seen in S3.

### Data analysis

The pre-decided criterion for removing outliers was to remove responses with latencies of less than 250ms, which are unlikely to be ‘true’ responses [13]. Response time data is generally highly positively skewed, but given that GLMMs are robust against non-parametric data [41], no further data trimming was planned. Planned analyses included two initial generalised linear mixed effect models (GLMMs), with accuracy and response times as outcomes respectively, Compatibility and Group size as fixed effects and Participant and Trial as random effects, to identify whether compatibility effects were to be found across all levels of Group size or whether there was an upper limit. For accuracy, binomial family and logit link function were used. For response times, gaussian family and identity link function were used. For all analyses, if models were singular, random effects structures were simplified by removing Participant then Trial.

Further planned analyses (only in the case of a significant interaction) included GLMMs to be carried out only on levels of Group Size where a significant compatibility effect was found in the initial stage of analysis. These would be structured with accuracy and/or response times as outcomes respectively, Distance (Compatible, Peripersonal and Extrapersonal) as the fixed effect and Participant and Trial as random effects.

Further analyses were conducted to investigate the possibility that there are two ways in which interference could occur in this task. Firstly, participants could code each individual partner’s location in the task, resulting in a response being triggered any time the finger points towards the self, and inhibiting a response when the finger points away (to any location). This was the assumption for our original planned analyses. However, it is possible that the presence of co-actors could more simply result in coding of the stimuli as ‘left’ or ‘right’, as has been suggested in the dyadic Joint Simon. This would mean a response would be triggered not only when the finger points directly towards the participant, but also when it points to their side of the screen. Additionally, this could be amplified by the arrangement of the participants within the group, depending on the number sitting on the left or right side of the screen. For example, if I am sitting on the right side, but there is no-one sitting on the left side (although there might be people sitting with me on the right side), co-representation based on left/right spatial coding should not occur. As such, we ran additional models on both RTs and Accuracy. To capture compatibility based on left/right coding of the stimuli, we firstly scored whether the finger pointed towards the participants’ side of the screen, and then whether this was compatible with the correct response for that participant (i.e., correct response is to respond, and the finger points towards the responder’s side of the screen). This formed our first fixed effect, Spatial Compatibility. We also added the difference between the number of participants sitting on the left and right side of the screen (ignoring those in the centre) as a fixed effect (Group Distribution - Screen), and Group Size. We excluded trials on which the finger pointed towards an empty chair.

Further, even if compatibility is triggered by the finger pointing towards me or away (i.e., regardless of the side of the screen I am sitting), there still could be an influence of the number of other players sitting to my right or left (rather than on the right or left side of the screen). For example, if I am sitting in the second seat from the right, and there is only one other player sitting on the first seat on the right, then I am the ‘left’ player even though I am sitting on the right of the screen. We carried out further analyses to test for the role of the number of players sitting on either side of the target player, by creating a variable ‘Group Distribution – Self’ consisting of the number of players to the target’s left minus those on the right. We then ran models with Compatibility (as originally defined), Group Distribution – Self and Group Size as fixed effects. We excluded trials on which the finger pointed towards an empty chair.

Additional data (age, sex, gender, ethnicity, first language, other languages, whether participants knew anyone in their group) were collected for exploratory analysis and to understand the generalisability of the sample. Using this data, for each trial, we generated the social relationship between target dyads (the correct responder and the recipient of the incompatible finger point) during incompatible trials. Firstly, we quantified whether the finger during incompatible trials was pointing at a familiar individual, a stranger, or an empty seat. Secondly, we calculated the number of peripersonal/extrapersonal positions available to the participant (e.g., if they were positioned in an edge seat, this would provide 1 Peripersonal and 3 Extrapersonal positions), to assess the possible influence of multiple direct physical neighbours. Finally, for those participants with two available peripersonal positions (i.e., those not sitting on an edge seat), we calculated the relationship to both adjacent participants (e.g., two familiar, one familiar and one stranger, two stranger etc.). For further exploratory analyses, we incorporated these social variables to assess the modulating effect of familiarity between physical neighbours and target dyads on accuracy and response time on incompatible trials. All analyses were carried out in JASP and R studio [42,43].

## Results

Datapoints from 28 trials with response times below 250ms were removed.

### Compatibility by Group Size

#### Accuracy.

**Fig 3:**
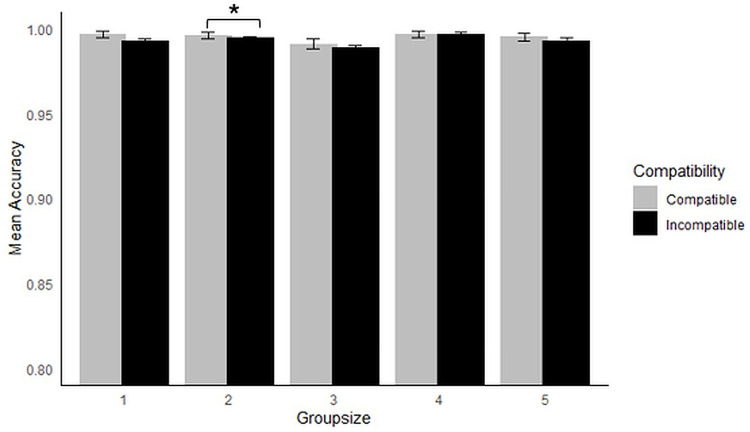
Mean accuracy by Compatibility and Group Size. Error bars show standard error.

Mean accuracy was close to ceiling across all conditions (see Fig 3). However, the GLMM showed a significant main effect of Compatibility (*X*^*2*^ (1) =  31.57, *p* < .001), Group size (*X*^*2*^ (4) =  33.32, *p* < .001) and an interaction (*X*^*2*^ (4) =  24.76, *p* < .001). Follow-up GLMMs were carried out on each level of Group Size separately, with Compatibility as a fixed effect and Participant and Trial as random effects, reducing random effects structures in cases of singularity or problems with maximum likelihood estimates. In cases where maximum likelihood issues could not be resolved by removing random effects, the model including all random effects is reported. The only significant Compatibility effect was found for groups of 2 (*X*^*2*^ (1) =  33.08, *p* < .001), with all other *p*s > .40.

#### Response times.

**Fig 4:**
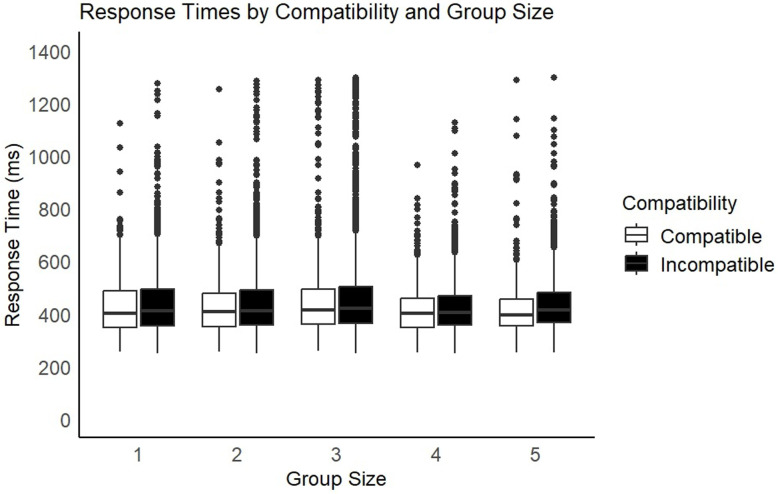
Mean correct response times by Compatibility and Group Size. Error bars show standard error.

The GLMM showed a main effect of Compatibility (*X*^*2*^ (1) =  35.62, *p* < .001) but no effect of Group Size and no interaction (See Fig 4).

### Distance

**Fig 5:**
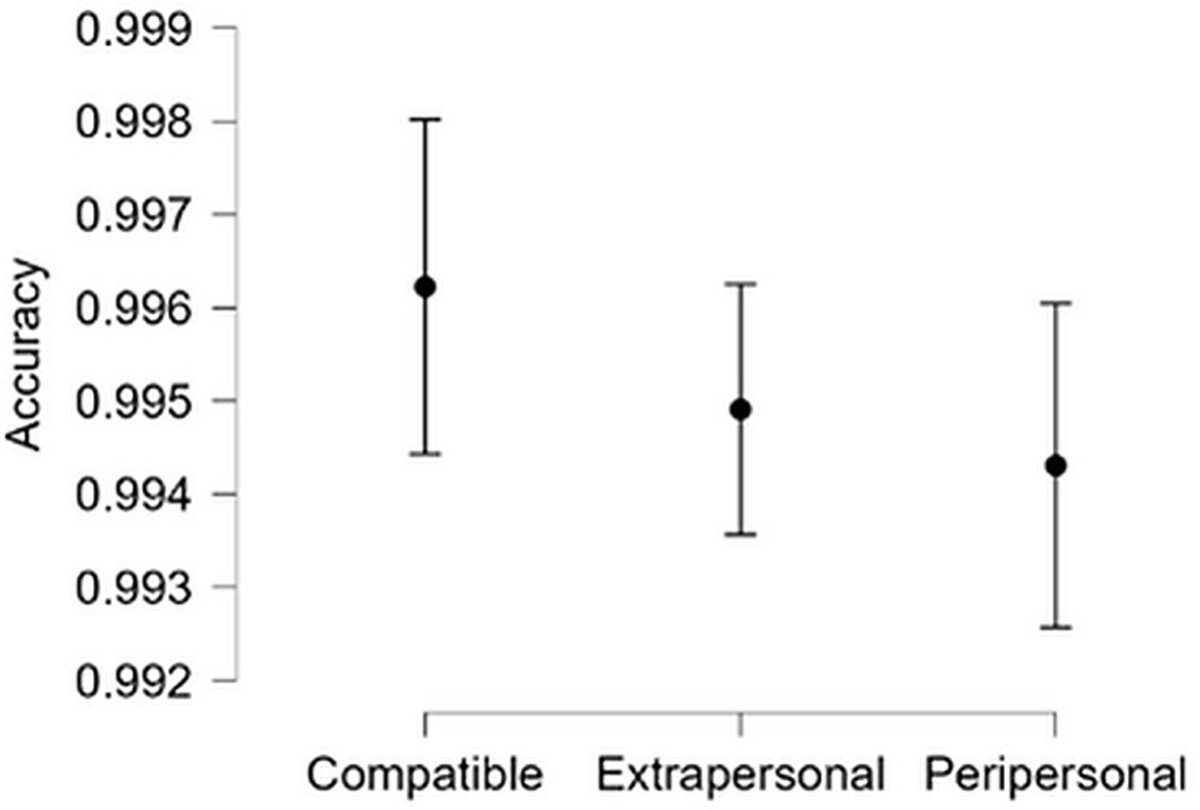
Mean accuracy for Groups of 2, by Compatibility (Incompatible condition split by distance). Error bars show standard error.

As the only significant compatibility effect was found for accuracy in groups of 2, for this subgroup, trials were broken further down into Compatible, Extrapersonal and Peripersonal. A GLMM found an overall effect of Distance (*X*^*2*^ (2) =  43.84, *p* < .001), but follow-up contrasts were not significant (all *p*s > .14, see Fig 5).

### Spatial coding: Participant’s location on left or right of screen

#### Accuracy.

Issues with maximum likelihood estimates could not be reduced by reducing random effects structures, so two separate models were carried out with 1) Spatial Compatibility and Group Size as fixed effects and Participant as a random effect and 2) Spatial Compatibility and Group Distribution – Screen as fixed effects and Participant as a random effect. Neither model showed any significant effects, and all models showed issues with maximum likelihood estimates.

#### Response times.

The same issues arose for RTs, resulting in the same two model structures as for Accuracy. No significant effects were present for either model.

### Spatial coding: Number of players to left or right of target participant

#### Accuracy.

Issues with maximum likelihood estimates could not be reduced by reducing random effects structures, so two separate models were carried out with 1) Spatial Compatibility and Group Size as fixed effects and Participant as a random effect and 2) Spatial Compatibility and Group Distribution – Screen as fixed effects and Participant as a random effect. Neither model showed any significant effects, and all models showed issues with maximum likelihood estimates.

#### Response times.

The full model was reduced to two separate models: 1) Compatibility, Group Size and Participant and 2) Compatibility, Group Distribution – Self and Participant, to reduce issues with maximum likelihood estimates. There was a significant effect of Compatibility for the second model only (*X*^*2*^ (1) =  20.65, *p* < .001), with no further effects or interactions.

### Familiarity effects on performance

The first exploratory GLMM (incompatible trials only, fixed effect: number of peripersonal positions, random effects: groupID, Participant, Trial) suggested that the response times of correct trials (but not accuracy) were impacted by the number of potential peripersonal positions available to the participants (i.e., whether they were sitting on the edge, with only one chair next to them, or in one of the three central positions, with two chairs either side). During peripersonal trials, the participant’s response time was slower when there was only a single peripersonal position to keep track of (compared with two, *β* =  −30.64, *se* =  12.05, *t* =  −2.54, *p* = .012; standardised *β* =  −0.27, 95% CI [−0.47, −0.06]), regardless of whether the position was occupied or empty.

**Fig 6:**
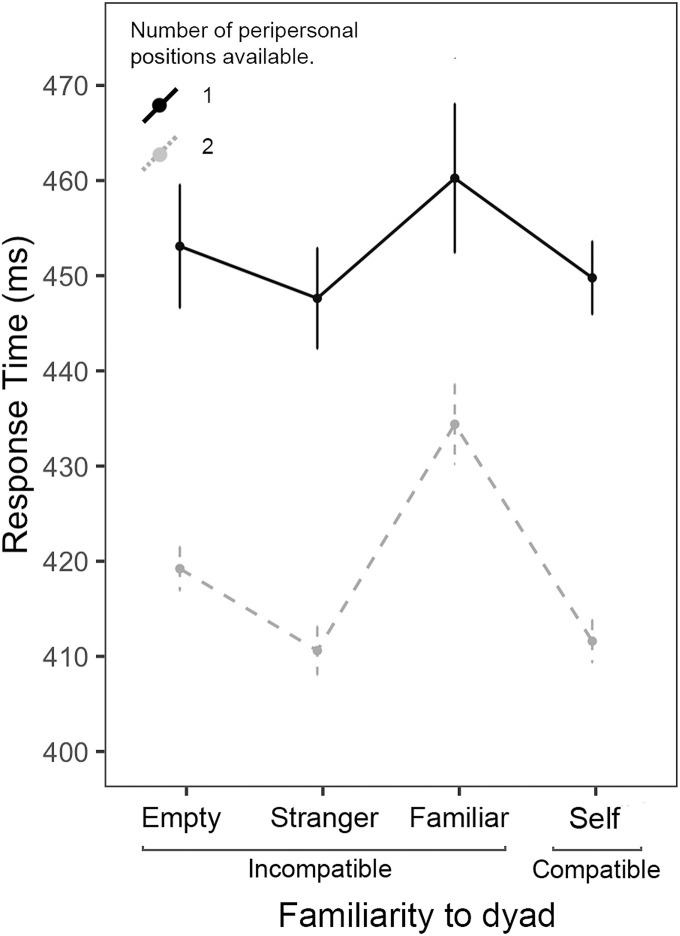
Mean response time during peripersonal trials, split into number of peripersonal positions available (1 or 2) and by familiarity to the dyad (familiar, stranger, empty seat or self (i.e., compatible trials). Error bars represent standard errors.

**Fig 7:**
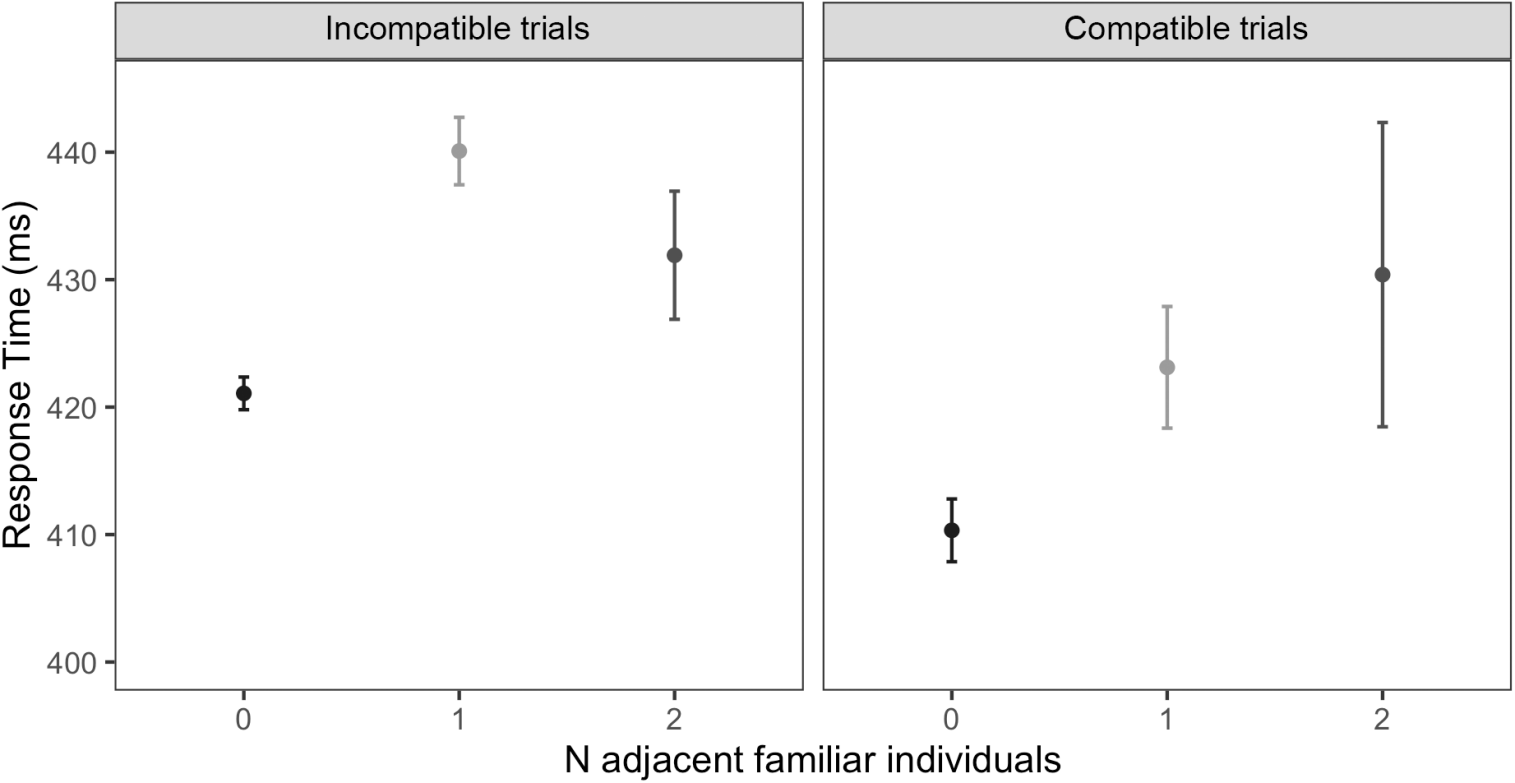
Mean response time during peripersonal trials with two peripersonal spaces, split into number of peripersonal familiar individuals present (0, 1 or 2). Data are split between incompatible and compatible trials. Error bars represent standard errors.

The second exploratory model (fixed effects: Familiarity (familiar, stranger, empty seat, or compatible) number of peripersonal positions; random effects: Group, Participant, Trial) suggested an impact of partner familiarity on response time. Specifically, responses during trials featuring a familiar incompatible partner were significantly slower than response times during compatible trials (Fig 6, *β* =  −11.05, *se* =  4.24, *t* =  −2.60, *p* = .009); standardised *β* =  -.09, 95% CI [-.17,-.02]). For those participants with multiple peripersonal positions (i.e., those *not* seated at an edge seat), the number of peripersonal familiar individuals impacted the response time during correct trials (but not accuracy). Being seated next to a single familiar individual (compared with no familiar individuals) significantly slowed response times during incompatible trials (Fig 7, *β* =  29.68, *se* =  14.72, *t* =  2.02, *p* = .046), but not during compatible trials (*β* =  13.16, *se* =  15.05, *t* =  0.88, *p* = .38). Being seated next to two familiar individuals however, showed no such effect. Furthermore, a significant compatibility effect was found during trials with one perpersonal familiar individual (*β* =  −16.45, *se* =  4.42, *t* =  −3.72, *p* < .001), but no compatibility effect was found for trials with two peripersonal familiar individuals (*β* =  −2.16, *se* =  10.37, *t* =  -.209, *p* = .84).

**Fig 8:**
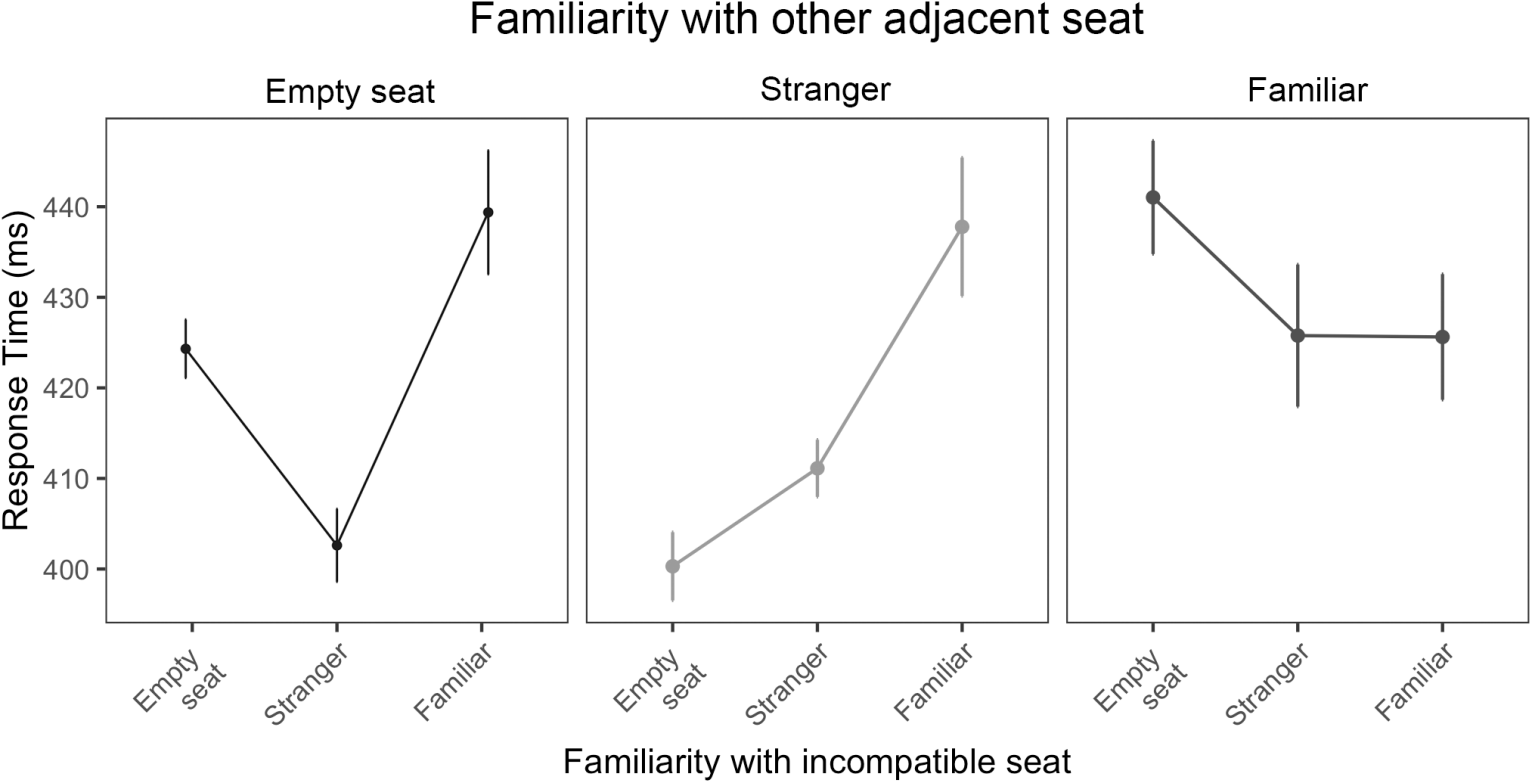
Mean response time during peripersonal trials with two adjacent spaces, split into the relationship with the incompatible target seat (empty, stranger, familiar) by the relationship with the other adjacent seat. Error bars represent standard errors.

This was broken down further to explore how the relationship to both peripersonal participants (the incompatible position, and the other adjacent position) impacted performance (Fig 8). When the adjacent, non-target seat was empty, responses were slower on trials with a familiar incompatible target compared with a stranger incompatible target. This was trending, but not significant (*β* =  37.68, *se* =  21.41, *t* =  1.76, *p* = .08). Further, when there was a stranger in the adjacent, non-target seat, responses were slower when the incompatible target was familiar, compared with an empty seat. Again, this was trending but not significant (*β* =  37.95, *se* =  19.94, *t* =  1.90, *p* = .06). The response times appear higher when a familiar individual was in an adjacent seat and the incompatible target was a stranger or empty (and lower when both incompatible targets and adjacent seats were both familiar). However, these data must be taken with caution and further research is needed to confirm.

No effects outlined in this section were present for accuracy data. We suspect this is likely due to the ceiling effects of the accuracy data, and thus lower variability to explore.

### Discussion

This study tested whether interference effects in a Joint Simon task could be elicited beyond dyadic settings and whether there is a limit on the number of partners that can be co-represented. It tested whether interference was dependent on being within a partner’s peripersonal space or not, helping to distinguish between Referential Coding [32] and Co-representation [2] accounts. Finally, it explored the influence of inter-individual relationships (specifically familiarity) between individual members within a group, to identify possible mechanisms for selectively tracking specific group members over others, when the number exceeds one’s capacity to track everyone.

Firstly, the results provided tentative evidence for a limit on co-representation beyond the dyad. Accuracy scores were significantly higher on compatible than incompatible trials, but only in groups of 2. No such interference effects were found in the individual control condition, nor in the larger group conditions (between 3–5 co-actors). This is the first evidence that co-representation may be limited by the number of co-actors present. It further suggests that the limit is much lower than what we see in recursive mindreading and natural conversation, indicating that the mechanisms are likely different. This result should be treated with caution, given the ceiling effects in accuracy, and the lack of similar effects in response times. Additionally, pilot research carried out by the first author [44], found preliminary evidence for co-representation in groups of 3. For this reason, it is important to replicate this work, ideally using a more difficult task that provides more variation in accuracy scores.

It is possible that this limit is variable, depending on the context. For example, cues that have been found to influence the presence or strength of co-representation in previous dyadic studies (e.g., distance [9], competition [38], group membership [37] etc.) could result in more or fewer group members being tracked, depending on the make-up of the group. For example, if a group is made up of close friends, co-representation might extend to a greater number of co-actors than a group made up of strangers. Alternatively, we might see co-representation of a greater number of co-actors in a context where participants are instructed to collaborate, as opposed to the minimally joint context inherent in the Joint Simon task. The current set-up may not have been optimal for identifying such effects, as it did not instruct participants to collaborate, and groups were mostly made up of a mixture of friends and strangers from contexts that were explicitly chosen to increase diversity (i.e., local council events rather than undergraduate participant pools). An important area for future study will be to investigate the interaction of these cues and determine how the associated underlying cognitive mechanisms (e.g., joint goals, attentional processes) function in complex settings. Although many of these factors have been studied in dyadic settings, this paradigm allows us to investigate the interaction between factors within the same group.

Secondly, when we broke down the effect of Compatibility in groups of 2 into compatible, incompatible (peripersonal) and incompatible (extrapersonal), we found a significant overall effect, but follow-up contrasts were not significant. This leaves open the question of whether interference is dependent on physical proximity. Descriptive statistics suggest the highest scores (and therefore least interference) for compatible trials, followed by extrapersonal, then peripersonal, which would suggest that interference occurs for all group members (even those far away), but more so for those closest to the participant. This would suggest that distance does act as a cue triggering co-representation in settings where a joint goal is lacking, but that other, possibly social cues, can result in co-representation even when a partner is acting at a distance. However, this interpretation should be treated as speculative, and replications should be carried out.

Further, we investigated the role of the spatial distribution of the group members. We first investigated the possibility that compatibility may be coded in reference to the side of the screen on which the target actor is sitting, relative to the side towards which the finger is pointing. Analyses based on this reference point found no effects of compatibility, nor any interaction with the number of participants sitting on the left or right side of the screen. These models should be interpreted with caution, given the persistent issues with maximum likelihood estimates. However, it highlights the importance of considering the reference point that may underlie co-representation in non-dyadic settings. Additionally, we ran analyses using our original reference point for compatibility (i.e., compatible =  pointing towards me, incompatible =  pointing away), but added a variable encoding the spatial distribution of the participants (number sitting to the left compared to right of the participant). We only found one significant effect of Compatibility on Response Times, but this was not consistent across all models.

Finally, exploratory analyses investigated the role of familiarity between individual members within the group and the number of potential peripersonal neighbours to be tracked. This was to investigate whether these could be factors that influence ‘selection’ of individual group members to track, when the number exceeds one’s cognitive capacity. We did this for target dyads (the correct responder and the recipient of the incompatible finger point) and for direct physical neighbours. We found that participants who only had one potential peripersonal neighbour (“edge-seaters”) responded more slowly on incompatible trials compared to those with two peripersonal neighbours (“central-seaters”). This could be explained either by perceptual differences in these participants’ view of the screen and the arrows (i.e., edge-seaters are viewing the screen from an angle), by interference resulting from experiencing fewer peripersonal decoy arrows (regardless of whether there is a peripersonal co-actor present), or by interference caused by tracking one partner but not two. The fact that familiarity with one’s direct neighbour caused additional interference renders the former two non-social explanations unlikely. Edge-seaters with a familiar direct neighbour were the slowest to respond overall, suggesting that the social relationship between co-actors plays a role in the extent to which they are tracked. If edge-seaters were merely performing worse on incompatible trials than central-seaters because they were less able to see the screen, then we should not expect the identity of the person responding on that trial to have any effect. Further, the same reduction in performance should be true for compatible trials, which was not the case. We propose that the latter, social explanation is therefore more likely, which would support the conclusion that co-representation breaks down when there is more than one co-actor to track.

Familiarity was important even when the occupant of the adjacent, non-target seat (for central-seaters) was taken into account. When one of the adjacent seats was empty, participants were slower when the decoy finger pointed towards a familiar direct neighbour, compared with a stranger direct neighbour. When a stranger sat in the adjacent seat, responses were slower on trials where the decoy finger pointed towards a familiar direct neighbour than to an empty seat or a stranger. This replicates previous findings highlighting the importance of social relationships in joint task interference [6,34–38]. The pattern was different when a familiar partner was sitting in the adjacent seat. On those trials, having a familiar, stranger or empty target seat did not make a significant difference, although means were slowest for an empty seat. This is difficult to interpret, but one explanation is that when there is more than one co-actor present, co-representation still occurs when there is a clear reason to prioritise one over the other (e.g., when one is a stranger and the other is familiar). However, when both co-actors are equally salient (e.g., both familiar), co-representation breaks down. It should be noted that these analyses were exploratory, and data were only approaching significance, so they need to be approached with caution. Further research could explicitly manipulate the relationship between group members to test this hypothesis.

Additional support for the conclusion that co-representation is limited to 2 co-actors comes from our finding that a compatibility effect existed when participants were seated next to one familiar individual but disappeared when seated next to two familiar individuals. This was regardless of whether the familiar individual was the incompatible target or the adjacent non-target. This fits best with the first of our two proposed mechanisms for how co-representation plays out in larger groups: namely, that when the number of co-actors exceeds one’s cognitive capacity, co-representation simply breaks down completely.

One feature of testing multiple naïve participants on a task such as this, is that it is not possible to maintain the equal number of compatible and incompatible trials that we see in dyadic tasks. This is because, whereas in dyadic tasks, incompatible trials always correspond to a single partner (i.e., half of the trials pointing towards them, half towards me), in larger groups, we need an equal number of incompatible trials for each of the group members (e.g., 1/3 pointing towards me, 1/3 to partner A, 1/3 towards partner B). We used statistical methods that account for unequal numbers of trials in each condition, but it is possible that the mere fact that participants are not expected to respond as frequently could influence (and possibly weaken) their tendency to co-represent. It would be worth investigating this further, possibly by manipulating the ratio of incompatible/compatible trials in a dyadic task to see whether this influences interference.

This study provides the first evidence for the Joint Simon effect in groups of more than two. It provides preliminary evidence that co-representation may break down in groups of three or more, which would mean that this mechanism cannot explain coordination in groups. Evidence for the role of physical proximity was ambiguous, given the non-significance of post-hoc contrasts. Importantly, exploratory analyses provide novel hypotheses regarding the mediating factors that may influence one’s capacity for co-representing more than one partner. Early indications suggest that there may be factors, such as familiarity, which allow selective tracking of individuals within a group who are more salient or relevant. This has implications for theoretical accounts of co-representation, demonstrating that this mechanism may be more cognitively demanding than previously thought. Further, it highlights the importance of investigating social cognitive mechanisms outside of dyadic settings to uncover the true complexity of our day-to-day social interactions.

## Supporting information

S1 Data Distribution of individual participants across Group Size condition and testing locations. (DOCX)

S2 Data Self-described participant ethnicity. (DOCX)

S3 Data Task instructions. (DOCX)
