# Supplementary material for: Co-representation breaks down beyond the dyad in UK adults
Source: PLoS One. 2025 Feb 25;20(2):e0318545. doi: 10.1371/journal.pone.0318545 (PMC11856543; doi:10.1371/journal.pone.0318545)
Supplement: S1 Data — (DOCX) [file pone.0318545.s001.docx]

**S1.** *Distribution of individual participants across Group Size condition and testing locations.*

| Group Size | Event | Lab | Total |
| --- | --- | --- | --- |
| 1 | 23 | 10 | 33 |
| 2 | 24 | 26 | 50 |
| 3 | 22 | 24 | 46 |
| 4 | 19 | 20 | 39 |
| 5 | 15 | 20 | 35 |
| Total | 103 | 100 | 203 |
