# Supplementary material for: Co-representation breaks down beyond the dyad in UK adults
Source: PLoS One. 2025 Feb 25;20(2):e0318545. doi: 10.1371/journal.pone.0318545 (PMC11856543; doi:10.1371/journal.pone.0318545)
Supplement: S2 Data — (DOCX) [file pone.0318545.s002.docx]

**S2.** *Self-described participant ethnicity*

| Description | N |
| --- | --- |
| Argentinian/Latin | 1 |
| Asian | 5 |
| Asian-Indian | 1 |
| Bangladeshi | 2 |
| Black African | 2 |
| Black British (African) | 1 |
| British | 17 |
| British Asian | 1 |
| British Indian | 1 |
| British Pakistani | 1 |
| Caucasian | 1 |
| Caucasian/German | 1 |
| Chinese | 6 |
| Cypriot | 1 |
| English | 3 |
| Filipino | 1 |
| Greek | 2 |
| Indian | 10 |
| Indian/Asian | 1 |
| Lithuanian/other white | 1 |
| Macedonian | 1 |
| Malaysian Asian | 1 |
| Mixed White Asian | 1 |
| Nigerian | 1 |
| Polish | 1 |
| Thai | 1 |
| White | 47 |
| White Italian | 1 |
| White/African | 1 |
| White/Chinese | 1 |
| White/Asian | 1 |
| White/British | 73 |
| White/European | 2 |
| Undisclosed | 12 |
