# Supplementary material for: Co-representation breaks down beyond the dyad in UK adults
Source: PLoS One. 2025 Feb 25;20(2):e0318545. doi: 10.1371/journal.pone.0318545 (PMC11856543; doi:10.1371/journal.pone.0318545)
Supplement: S3 Data — (DOCX) [file pone.0318545.s003.docx]

**S3.** *Task instructions*

In a moment you will see a hand appear on the screen. The hand will appear over and over again and it could be pointing in one of several different directions. The hand will have a ring on its finger, which will be one of the colours you see in front of you [point to coloured paper on floor]. Your job is to press your button as FAST as you can whenever the ring is your colour – the one you see in front of you [point to all colours]. You will also see some coloured dots at the bottom of the screen, which correspond to these colours [point to colours on floor]. You can completely ignore these – it doesn’t matter which colour the finger is pointing towards, you ONLY press your button when the RING is your colour. First we will have a practice.

[Run practice]

Great, any questions? Then we can get started on the real thing. There will be 3 blocks of about 5 minutes each, with a break in between each one. When the break comes up, just give me a shout and I will get the next block running when you are ready.

[Run task]
